# School Nurses’ Experience of Working in School Health Service during the COVID-19 Pandemic in Sweden

**DOI:** 10.3390/ijerph18136713

**Published:** 2021-06-22

**Authors:** Eva Martinsson, Pernilla Garmy, Eva-Lena Einberg

**Affiliations:** 1 Faculty of Health Sciences, Kristianstad University, 29188 Kristianstad, Sweden; evamartinsson7@gmail.com (E.M.); pernilla.garmy@hkr.se (P.G.); 2 Clinical Health Promotion Centre, Lund University, 22100 Lund, Sweden

**Keywords:** school nurses, school health service, COVID-19 pandemic, focus group interviews

## Abstract

The COVID-19 pandemic has had a vast influence on Swedish society. Related recommendations and political decisions have greatly affected schools. This study aimed to describe school nurses’ experience working in Sweden during the pandemic in 2020. The study used a qualitative method with an inductive approach. Interviews with 17 school nurses in five focus groups and one individual interview were conducted. Qualitative content analysis was used. The impact of the pandemic on school nurses can be described through three categories: “Changes in working methods in relation to the students/guardians”, “Impact on cooperation with school staff”, and “The school nurse’s prerequisites for major changes.” Overall, school nurses experienced a transition to a digital way of working. Policies and decisions on global and local levels affected the work situations of school nurses as well as the school nurses’ social, cultural, and professional experience. The highest priority for school nurses is students, and school nurses adapted their working methods to give support to students during the changing circumstances. School nurses are both pragmatic and highly creative. Cooperation with other school professions is critical, as is support and guidance during crisis situations.

## 1. Introduction

### 1.1. Background

In the Ottawa Charter of 1986, the World Health Organization (WHO) described health promotion as a process that allows people with the right guidance to participate in and improve their state of health [1]. The school nurse has an important role in the work of the school health service: to promote students’ health and learning. The School Act [2] states that elementary and upper secondary school students should have school health services that include medical, psychological, psychosocial, and special pedagogical measures focused on prevention and health promotion that support students’ development toward the objectives of education. Health dialogues or health conversations are among the school nurse’s most important tools in health promotion work. In the health dialogues, diet, sleep, exercise, and stress management are central. Golsäter’s dissertation [3] delves into health dialogues and emphasizes the importance of preparation for the student and adaptability from the school nurse. The school nurse’s health promotion work is carried out at both group and individual levels [4].

Morberg highlights some important aspects of the school nurse’s professional conditions. Teamwork with other professionals in the school health service plays a key role. Working alone, as school nurses do, can render a sense of uncertainty and vulnerability but also a sense of control and the ability to work independently. The role of school nurses in relation to a school’s core, which is teaching and learning, can mean difficulties for nurses in asserting their professional roles [4]. The school health service in Sweden consists of the principal, special education teacher, psychologist, counselor, school nurse, school physician, and study and career counselor. The teamwork between the school health service and the pedagogical staff contributes to providing good learning environments and creating good learning situations for the school’s students [5].

The school nurse’s professional role in relation to the teamwork in the school can be described and understood based on the theory of capital, habitus, and fields of the French sociologist Pierre Bourdieu [6]. Capital is about different types of resources people bring with them, such as socio-economic background, education, and professional experience. Capital is valued by others and has an impact on the position a person acquires in social contexts [7]. Bourdieu also uses the term “habitus,” which is linked to past experiences people bring, such as values, attitudes, traditions, and social structures, that affect how they think and interpret reality [7]. A person’s capital can be social, cultural, and symbolic and is also something that is relational in a field such as the school [6]. The term “field” is used by Bourdieu for social fields in different areas. The school can be seen as a field. The school nurse in that field does not have the capital that is directly useful for the school’s mission, i.e., education and teaching. Morberg [4] points out that the school nurse’s habitus and capital depend on how other professions perceive their value to the school. In turn, this affects the legitimacy of the school nurse, which reflects how useful her area of knowledge is at the school.

When the coronavirus had reached all continents, the WHO on March 11, 2020 declared it a pandemic [8]. Different countries chose various strategies to reduce the spread of infection. Many chose lock down, i.e., a shutdown of the whole society with different levels of isolation and for different lengths of time, which affected mental health to a large extent [8]. By March 18, 107 countries had opted for national school closures, affecting about 862 million children and young people, which is equivalent to half of the world’s students [9]. In an overview article, Viner et al. [9] examine previous research on school closures, e.g., in connection with SARS and MERS. Some studies conclude that closure has some effect; others conclude that it has very little effect. Sweden’s COVID-19 management during spring 2020 differed from that of many other countries. The reasons why Sweden chose to use softer governance could be both due to the high trust that exists within Sweden for both people and authorities but also due to legal aspects. There were legal obstacles for the government in making decisions about lockdown [10]. Sweden chose to keep schools open. “Keeping schools open was an active strategy in Sweden to meet the threats of the COVID-19 pandemic” [11] (p. 503). Lindblad et al. argue that education becomes a conscious strategy in the fight against the COVID-19 pandemic and in keeping society going [11]. The strategy for the spread of infection was to slow it down, not stop it [12]. During the spring, Sweden was heavily criticized internationally for its strategy [13]. International research on the subject is ongoing, but some short posts show that the school nurse in many countries played an important role in students’ well-being during the pandemic and in many cases also during school closures [14,15,16].

During the ongoing COVID-19 pandemic, the Public Health Agency of Sweden stayed in consultation with the government, which provided information on pandemic practices. The recommendations were as follows: wash your hands, keep your distance, avoid larger parties and unnecessary travel, and stay at home if you are ill [17]. It was up to each school and workplace to ensure compliance. At the same time, it was up to each individual, including children and young people, to adhere to the rules to reduce the spread of infection. The school nurse was able to provide support to those who felt stress and anxiety.

In Sweden, some research has been conducted and reports written on distance learning during the COVID-19 pandemic [18,19,20], but there has been no published research on the school nurse’s work during the same period. Both school nurses and managers can benefit from learning how the work situation of a specific professional group can be affected when an extraordinary situation arises in society. This study will contribute to highlighting how the work of school nurses was affected during the COVID-19 pandemic.

### 1.2. Aim

The aim is to highlight school nurses’ experience of their work situation during the COVID-19 pandemic in Sweden.

## 2. Materials and Methods

### 2.1. Design

The study applied a qualitative method in which data were collected via digital interviews in focus groups and one individual interview. Interviews are suitable for examining new or complex topics but also for examining people’s experiences [21]. Qualitative research is used to investigate people’s perceptions, experiences, and meaning in relation to a particular phenomenon [22]. Elo and Kyngäs [23] recommend inductive content analysis when few studies have been done and only fragments of knowledge exist about a phenomenon. Before the recruitment of informants, a research ethics application was approved by the Lund Regional Ethics Review Board (EPN 2018/842; 2020-04871).

### 2.2. Settings and Sample

Data collection was conducted from the end of November 2020 to January 2021 in Sweden. The context of the pandemic changed during this period, and the changes have been noted in the study. This study used purposive sampling, which aims to select people who can provide informational descriptions of what is being investigated [22]. The sample consisted of school nurses who worked with students aged six to 19 in Sweden throughout 2020. All the participants were women. The school nurses’ work experience ranged from 1.5 to 20 years. A school nurse in Sweden has a post-graduate specialist nurse education, including public health and child/adolescent health care [24]. The number of students the school nurses were responsible for varied from 100 to 600, and the degree of service varied among the school nurses. They worked with students from different socio-economic contexts; among the students were immigrants as well as students with neurodevelopmental disorders (NDDs).

### 2.3. Data Collection

Initially, letters with information about the study were sent to the central school health administration in 12 municipalities, and six of them agreed to participate. Schools in both rural and urban areas were represented. Both private and public schools and both elementary and upper secondary schools were represented. To provide a variation, municipalities with low as well as high prevalence of inhabitants with COVID-19 were selected. In the six municipalities that agreed to participate, the information was then distributed to the school nurses, who were given contact information for the researchers. Sixteen school nurses contacted the first author and were included in the study. The sample was still considered too low, and in the next step, information about the study was posted to a manager in a private company for Swedish school nurses, and this provided an additional three respondents. In summary, 19 school nurses agreed to participate; however, two school nurses informed the researchers that they could not participate because of a heavy workload (*n* = 1) or health problems (*n* = 1). The goal was to conduct focus group interviews with three to four participants in each group. This was the case in four of the six interviews; see Table 1. In two focus group interviews, participants informed the researchers that they could not participate with short notice. However, other participants who wanted to take part in the focus group interview had already arrived; therefore, in one case, the focus group interview was conducted with two school nurses, and another case was conducted as an individual interview. The individual interview and the focus group interview with two nurses provided rich data, which also applied to the other focus group interviews, and thus, all interviews were included in the analysis. Finally, the result includes 17 school nurses.

Data were collected with digital interviews via Zoom, as workplaces restricted in-person meetings. The first author acted as a moderator and the last author acted as an observer in all five focus group interviews. The individual interview was conducted by the first author. The interviews were semi-structured and began with an open question, proceeding to follow-up questions to get in-depth responses. An example of the questions: “Please tell me about your experience of working during the COVID-19 pandemic.” The interview guide used was discussed after the first focus group interview was completed. It was considered to work well except for minor adjustments according to the order in which the questions would be asked. Each focus group interview took about one hour, and the individual interview lasted about 45 min. One purpose of the focus group interviews is to understand collective experiences [25]. The school nurses who participated spoke freely and with great commitment about their experiences. The permissive interview climate brought out differences and varied viewpoints. The benefit of using digital focus group interviews in this study was that geographical distances were not an obstacle [26].

### 2.4. Data Analysis

The analysis was based on Elo and Kyngä’s [23] inductive content analysis. After transcription, analytical units were selected. The process of analysis featured moving back and forth between the whole and the analytical units. This included listening to the recorded material and the reading of the entire body of transcribed material. After that, the organizing phase began, with open encoding, encoding sheeting, grouping, and categorization. The initial analysis was conducted by the first author. Then, the first and the third author continued the analysis process, thereby identifying similarities and differences. In the last step, the second author was involved in the analysis process. The preliminary results have also been discussed at seminars during the analysis process. After discussions between all authors, consensus was reached. For an overview of the analysis process, see Table 2. The results consist of three categories with a common thread as an overall level.

## 3. Results

The aim of the study was to highlight school nurses’ experiences of their work during the COVID-19 pandemic. The main results that emerged were the following three categories:

- Changes in working methods in relation to students/guardians;
- Impact on cooperation with school staff;
- The school nurse’s prerequisites for major changes.

**Figure 1:**
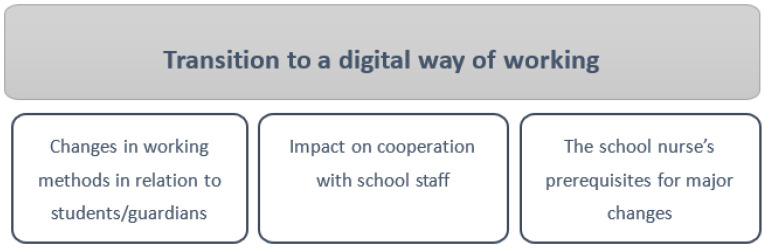
A common thread, “Transition to a digital way of working,” and three categories: Changes in working methods in relation to students/guardians, Impact on cooperation with school staff, and School nurses’ prerequisites for major changes.

A common thread that was consistent in all three categories and can be said to be on an overall level is the transition to a digital way of working; see Figure 1. 

### 3.1. Changes in Working Methods in Relation to Students/Guardians

The school nurses expressed that the recommendation from the Public Health Agency for everyone to stay at home when you are ill was largely followed by both students and staff at both elementary and upper secondary schools. According to the school nurses, many students stayed home the first week that the recommendation was introduced, but then absenteeism gradually decreased.

According to the school nurses, when students worked from home, the work changed in a few ways, including health dialogues moving to a digital platform, with school nurses making phone calls and having chats and often digital video dialogues. There were mixed experiences among school nurses about the digital health dialogues. One expressed, “I think it works so badly. It kind of does not become this atmosphere where you can smoothly transition to a little more sensitive topic” (F 4). One school nurse found it difficult working with the students who felt unwell and sought contact via chat, “Those who feel really bad, who would need to have conversations. So, I think it’s much harder” (F4). Others thought it worked beyond expectations. “It is perfectly okay to conduct a digital health dialogue with the student in the picture” (F 2). One highlighted that web meetings using a webcam are helpful, because then you see what the students look like and how they take care of themselves.

New things arose to consider, such as checking to see if the student was alone so that sensitive topics could be dealt with. In some areas, the school nurse gained new experiences. Many school nurses gained greater insight into their students’ home situations, both good and bad. Others pointed out that it was easier to reach guardians, as many of them also worked at home.

Several school nurses pointed out that they had many more health dialogues when offering digital call-in for parents and guardians, who were allowed to participate remotely but not on site. This meant a time gain for the school nurse and other participants in meetings that involved both students and parents. Having health dialogues digitally in certain situations will probably be an opportunity in the future. One school nurse took her own initiative based on the Public Health Agency’s general recommendations to have outside “walk and talk” health dialogues.

Some school nurses described that they, due to the COVID-19 pandemic, had recorded informational videos about health to reach out to the students; this is another practice that could be continued post-pandemic. In the focus group discussions, the nurses noted that prerecorded videos provided an opportunity for students to go back and look at what was said about, for example, sleep, instead of just hearing the information once. Another discussion was that students are already on digital platforms and that school health service staff could approach them there.

For school nurses, there were more conversations about serious topics. When talking to the students, nurses saw that they had gained a greater understanding of what the pandemic meant. Some had suffered losses. “We have been in conversations about grief and loss and then they know someone who has lost someone and that means that the seriousness of it all has increased, and the insight into it” (F 2).

A new task for the school nurse was to convey restrictions imposed by schools based on Public Health Agency recommendations; this was primarily difficult in dealing with guardians from different linguistic backgrounds.

One school nurse was committed to providing a traditional graduation for those who completed school in the spring when the restrictions prohibited gatherings of more than 50 persons. Planning it was high-level logistics. A school nurse highlighted that it was a matter of being able to switch between old on-site routines and finding ways to work in the digital realm.

### 3.2. Impact on Cooperation with School Staff

The school nurse is part of the school health service, which is the group the nurse works with most closely. Several school nurses mentioned that internal meetings at the school took place digitally, which affected school health service meetings. Among the school nurses there were differences of experience about whether digital meetings were good or bad. “I think it has been possible to have good meetings with school health service; you can solve things smoothly digitally” (F 4). One school nurse did not think that the digital school health service meetings worked well and said that could have an impact on students in the longer term because information shared digitally is not as detailed.

It emerged that cooperation increased within the school health service at upper secondary schools during the period of distance learning and that there was a focus on supporting students who had difficulty with this type of education. The teachers also played an important role, and cooperation with teachers was important for school health service.

The school nurses said they and school staff had closer contact with their students and got to know many more deeply when lessons were remote. “The fact that they called more often, they were called in sometimes… So it actually made quite great demands on the school health service during that time, but it was an enormous experience and you can lean on it now” (F 2). The school health service worked to get students who had difficulty keeping up to school so that they could pass their courses. Several noted that distance learning was difficult for students who were immigrants or had learning difficulties or neurodevelopmental disorders (NDD). A school nurse described it as “We [school health service] fought and fought before we got back the students during spring 2020” (F 4). Several school nurses pointed out that while working with students remotely, the school health service needed to be more active to keep track of how students were doing. The need for students to connect was great, as almost everyone missed seeing their friends.

The school nurses described that the government’s decision that upper secondary schools should operate remotely had different effects on the students, with sometimes unexpected results. Some schools achieved better attendance among students when they worked from home, and for some students, distance learning was a good fit. They have “been able to concentrate better when they have been at home and have better attendance” (F 5). The school nurses’ experience was that some students had difficulty with distance learning from their home computers. A lesson school management and school health service learned during spring distance learning was put to use in December, when it was again time for distance learning. “When they closed this time, they immediately announced who was exempt, and it was very clear” (F 4). There were exceptions for students in programs that had proved poor matches for distance learning in the spring, such as language introduction courses and four-year programs for students with neurodevelopmental disorders (NDDs).

After the summer, students returned to the school building. The school nurses discussed that some schools had trouble getting students to attend in person because of fears of COVID-19. “There was a lot of uncertainty with the parents and the students. There was high absence… before the distance, high absence due to corona” (F 5). Absences varied during the period and were different in upper secondary school and elementary school.

### 3.3. The School Nurse’s Prerequisites for Major Changes

This section focuses on the workplace and the school nurse in the organization. It emerged in the school nurses’ discussions that prerequisites meeting major changes were different in different organizations and workplaces. In some focus groups, it emerged that there were differences among the schools in terms of the support the school nurse received from her boss and from school nurse colleagues at other schools during the pandemic. For example, some schools introduced routines for announcing the number of students and staff who were infected, while others did not. There were also differences in different municipalities. School nurses who belonged to a central school health service organization experienced great support from management and received clear local directives about work procedures.

Support for the school nurses’ work also varied. Some school nurses had weekly meetings with managers and colleagues; others had such meetings less frequently. Some had good support from their managers and were told to follow documented routines for working practices during the COVID-19 pandemic. “We receive tremendous support from our manager regarding the pandemic” (F 3). Others felt they lacked support. Collegial support also varied. “We have a lot of Skype meetings with colleagues among each other” (F 3). Some school nurses lacked the ability to discuss the COVID-19 pandemic with management and colleagues to discern the best way to carry out their work.

The school nurses had different experiences of the Public Health Agency’s recommendation about working from home as much as possible. Whether school nurses did so looked very different depending on the manager’s attitude. At some schools, it was possible to do so. Other nurses did not have the opportunity to do so and pointed out that different staff groups at the school were treated differently.

The school nurses expressed that they had the opportunity to switch between being on site and working from home. Those who worked in several schools were instructed not to travel from school to school but to work one day at one school and the next day at another. In other municipalities, the school nurse was instructed to be on site together with others in school health service, to follow Public Health Agency recommendations, and to work in much the same way as usual. “We will just keep working as usual, we are having full receptions, meet every adult, every child, anything. We are exposed for this daily, without protection, no visor, no mouth guard” (F 3). There was concern among different staff groups who were at risk or had relatives at risk that they would have to continue working onsite even though the infection rate had increased.

The school nurses had ethical reflections on their pandemic work situations. This captured the difficulty of fully following the recommendations. One school nurse had a hard time resisting students who came to get a hug. “A lot of children who don’t get hugs at home, they come to just get a hug. … I’ve had a really hard time saying no. … I think that has been my biggest challenge with the social distancing among the younger children, who may not get this at home” (F1). The recommendation on social distancing was sometimes difficult to keep. Another expressed difficulty with the recommendations more generally, saying it was hard to know “what is right, which is wrong, where am I in these restrictions that exist?” (F 3).

## 4. Discussion

**Figure 2:**
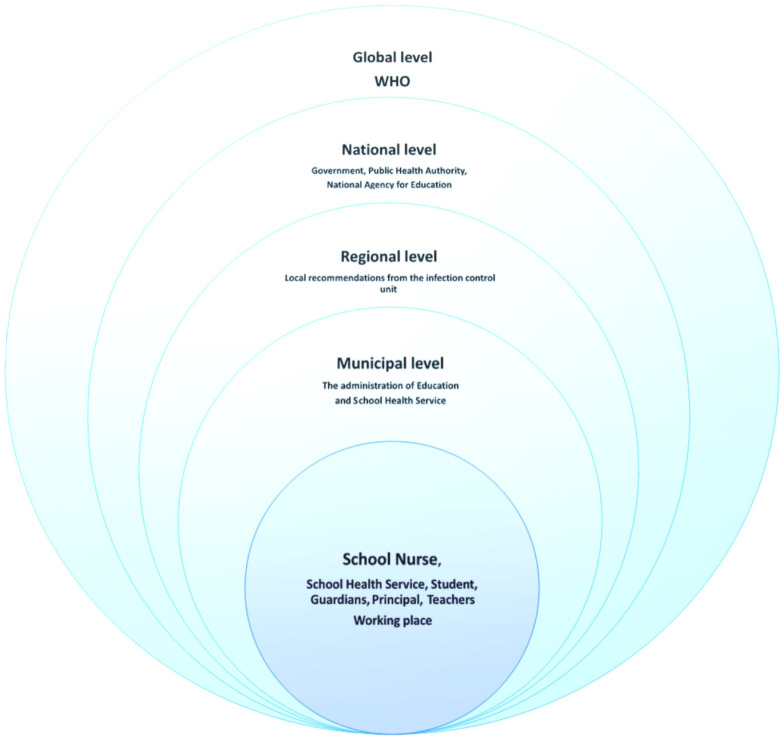
The school nurse’s work and decision-making levels, which affect work during the pandemic.

The work situation of school nurses is affected by both changes that take place in the outside world and the experiences the school nurse brings with her, the latter expressed in Bourdieu’s terms “habitus” and “capital”. Our point of departure for the performance discussion was the model below that was developed in this context; see Figure 2. It shows how the school nurse in her professional practice is affected by the outside world, where decisions at various levels affect what happens at the school nurse’s workplace. Based on the model of external influence on the school nurse’s situation, reasoning is carried out in relation to the three categories in the study’s results, while Bourdieu’s theory is woven in in terms of what the school nurse brings with her in education capital and experience. The work of the school nurse is affected by what happens at all the different levels.

The three categories of results cover the communication of school nurses at the individual, group, and organizational levels. The first category concerns changes in relation to the students and guardians. The Swedish National Agency for Education’s decisions on distance education vs. on-site teaching and the Public Health Agency’s recommendations on staying home affected attendance levels. School nurses had to shift from on-site availability to active availability by phone or other media. This is consistent with Morberg’s study [4], which emphasizes the importance of school nurse accessibility.

The Swedish National Agency for Education’s decision that teaching would take place remotely from the end of March provided a rapid transition that affected the way upper secondary school nurses worked, and of course also the way teachers worked [20]. Our interpretation, based on the results, is that school nurses are flexible, creative, and adapt to new situations in a pragmatic way based on decisions made at different levels. School nurses have moved relatively quickly to digital health dialogues with students. When the government made decisions about, e.g., restrictions on indoor training, some school nurses thought in new ways and gave advice on outdoor training that would meet the new restrictions. Innovation was also inspired by other people’s responses to the epidemic; for instance, the recorded lessons teachers made for students inspired some school nurses to record their own health videos. Not all school nurses respond in the same way, and here, based on Bourdieu’s theory, it can be seen that school nurses’ different habitus and capital influence how they act. Thus, the school nurses’ different experience capital affects their actions in a new situation.

The second result category highlights that the work with school health service teams is important. Apker et al. [27] present four different communication strategies that school nurses use when working in teams, the first being obtaining as much information as possible. The others are reliability, understanding, and coordination in order to achieve the best possible result. Our interpretation is that one of the school nurse’s fundamental tasks is to support students in different situations, which means that cooperation with other professions in school health service and teachers increases in a crisis situation. A complementary interpretation may be that the entire school health service and other school staff are mobilized in a crisis situation. The COVID-19 pandemic has the characteristics of a crisis according to crisis research [28,29,30,31]. Cooperation with the school health service intensified, primarily after the Swedish School Agency’s decision on teleworking in upper secondary school. The teamwork was about engaging students who had difficulty coping with school work remotely. This is reinforced by the report produced by the Swedish School Inspectorate, which is based on interviews with some 260 principals and highlights certain groups of students as very vulnerable during distance and remote education [32]. Bourdieu’s theory implies that the school nurse’s capital is relational and dependent on the value of the school’s specific context [6]. Increased cooperation with the different kinds of professionals working at school can change the value of the school nurse’s work. The teamwork also makes a transition to digital working methods within school health service, where decisions are made at the municipal or workplace level based on Public Health Agency recommendations on keeping distance.

The third result category, the school nurse’s prerequisites for major changes, highlights the school nurse in the organization and the way the school nurse is also dependent on management and school nurse colleagues. Our interpretation is that for a professional group with a lot of solo work, support is needed at work, as is the opportunity to discuss new situations that have arisen. Morberg [4] points out that solo work can create uncertainty and vulnerability when colleagues are not around. In a work situation, when old routines change very quickly, reactions can be very different depending on what experience and what other conditions in the form of habitus one has. Ljungblad and Näswall [33] note that social support from managers and colleagues reduced stress and ill health among employees, which they show applies in both acute situations and in the long term. In the crisis situation caused by the COVID-19 pandemic, with rapid transitions in the workplace, support was particularly important. Morberg et al. [6] point out that the habitus and capital of a school nurse is dependent on how members of other professions perceive the nurse’s value to the school. In turn, this affects the perception of the school nurse’s legitimacy, i.e., how useful her area of knowledge and profession is [6]. In the results, one school nurse highlighted that her medical knowledge was sought after and that her capital increased in relation to the other groups at the school during the COVID-19 pandemic. It is surprising that school nurses were not more in demand considering their expertise on communicable diseases. However, there may be various reasons for this, such as the fact that the room for maneuvering in the workplace depends on decisions at different levels, in this case national and municipal. An ethical question in this context is whether all school nurses should follow the same protocols or whether protocols should differ among schools and regions in a situation such as a pandemic. Morberg [4] points out that solo work provides autonomy in planning, managing, and carrying out work, but within certain frameworks. In an extraordinary situation, decisions are centralized so that as many people as possible act in the same or a similar way. This means less room for individuals to maneuver. Different levels sometimes clash, e.g., the Public Health Agency’s recommendation on social distancing clashes with the school nurse’s knowledge of children’s need for proximity—and at the same time, the school nurse has an awareness that she has not followed the recommendation.

### Strengths and Limitations

The first author served as moderator in all groups, and the last author was an observer in the five focus groups, which is a strength of the study. The observer summarized or collected threads that were dropped, and in some groups, additional thoughts emerged, which increases the reliability of the results [25]. The goal was to conduct focus group interviews with three to four participants in each group, and this was the case in four focus group interviews. However, because of dropout with short notice, one focus group consisted only of two school nurses, and one interview was individual. However, all the six interviews, conducted in groups, in pairs, and individually, worked well. A limitation may be that the focus groups had to be carried out digital, due to the pandemic, which may have affected the spontaneity of the discussions. Still, the narratives became rich. Furthermore, as there were differences between elementary and upper secondary schools, regarding restrictions related to the pandemic, it might have been better to focus on either school form. The Swedish school context may also restrict the transferability of the results to other contexts.

## 5. Conclusions

The work of the school nurse is influenced by decisions at different levels, from global to local, but also by what the school nurse brings in terms of professional experience and personal competence. School nurses prioritize working with students and adapt their way of working to support students based on conditions that are constantly changing. School nurses are both pragmatic and creative. Cooperation with coworkers in other professions at the school is of great importance. Support from both management and colleagues as well as the possibility of supervision for school nurses is needed in a crisis situation. The knowledge from this study can provide greater readiness for school nurses to deal with new situations that arise. If a similar situation should arise, management and human resources staff may use the study to understand the school nurse’s experiences during this period and to better plan the interventions and support that are needed from different directions.

## Figures and Tables

**Table 1:** Focus groups and individual interviews, number of participants, and workplaces.

Focus Group/Interview (Number)	Participants (*n*)	Workplaces
1	4	Elementary school (students aged 6–15 years)
2	3	Upper secondary school (students aged 16–19 years)
3	3	Elementary school (students aged 6–15 years)
4	2	Upper secondary school (students aged 16–19 years)
5	4	Upper secondary school (students aged 16–19 years)
6	1	Elementary school (students aged 6–15 years)

**Table 2:** An overview of the analysis process.

Analytical Units	Encoding	Grouping	Categorization
I had a few health dialogues over the phone and it went well, too	Health dialogues by phone	Changed way of working with health dialogues during the pandemic	Changes in working methods in relation to students/guardians
We met digitally with the school health service team every day, so we went through all the students and the teachers had to email us about which students they were worried about, who had not been to class, so that we actually had a better attendance during that time than when they go to school…	Digital meetings with professionals working in school	Cooperation digitally	Impact on cooperation with school staff
We get enormous support from our manager when it comes to the pandemic, where we have received restrictions on how to do, in terms of both visits, health visits, contact with other professional categories, everything as well, actually documented that we should just follow it	Support from the manager	Management and support guided the school nurse	The school nurse’s prerequisites for major changes

## Data Availability

The data presented in this study are available on request from the corresponding author. The data are not yet publicly available due to work in progress.
